# A Pilot Study to Assess Visual Vertigo in People with Persistent Postural–Perceptual Dizziness with a New Computer-Based Tool

**DOI:** 10.3390/jcm12051766

**Published:** 2023-02-22

**Authors:** Tsun-Ai Jasper Chen, Marie-Li Dion Parenteau, Kirby Marchand, Hong Zhi Zhang, Elizabeth Dannenbaum, Anouk Lamontagne, Joyce Fung

**Affiliations:** 1 School of Physical and Occupational Therapy, McGill University, 3654 Prom Sir-William-Osler, Montréal, QC H3G 1Y5, Canada; 2 Jewish Rehabilitation Hospital, Site of the Centre Intégré de Santé et de Services Sociaux de Laval (CISSS-Laval), Research Site of Centre de Recherche Interdisciplinaire en Réadaptation du Montréal Métropolitain (CRIR), Laval, QC H7V 1R2, Canada

**Keywords:** dizziness, vestibular, visual vertigo, persistent postural–perceptual dizziness, assessment

## Abstract

Background: Visual vertigo (VV) is a common symptom in people with persistent postural–perceptual dizziness (PPPD). Few subjective scales are validated for assessing the intensity of VV, yet these scales are limited by recall bias, as they require individuals to rate their symptoms from memory. The computer-Visual Vertigo Analogue Scale (c-VVAS) was developed by adapting five scenarios from the original paper-VVAS (p-VVAS) into 30 s video clips. The aim of this pilot study was to develop and test a computerized video-based tool for the assessment of visual vertigo in people with PPPD. Methods: PPPD participants (*n* = 8) and age- and sex-matched controls (*n* = 8) completed the traditional p-VVAS and the c-VVAS. A questionnaire about their experiences using the c-VVAS was completed by all participants. Results: There was a significant difference between the c-VVAS scores from the PPPD and the control group (Mann–Whitney, *p* < 0.05). The correlation between the total c-VVAS scores and the total c-VVAS scores was not significant (r = 0.668, *p* = 0.07). The study showed a high acceptance rate of the c-VVAS by participants (mean = 91.74%). Conclusion: This pilot study found that the c-VVAS can distinguish PPPD subjects from healthy controls and that it was well-received by all participants.

## 1. Introduction

Persistent postural–perceptual dizziness (PPPD) is a chronic vestibular disorder characterized by persistent, non-spinning dizziness, visual/motion hypersensitivity, and perceived unsteadiness [1]. Due to the variety of symptoms, PPPD has physical, psychological, and social consequences. Those with PPPD commonly experience exacerbation of symptoms in environments with complex and moving visual stimuli, such as shopping centers, grocery stores, and busy intersections [2,3,4]. They may also have difficulty watching TV and movies. As there are no established physical, laboratory, or imaging findings, the diagnosis of PPPD is made from clinical judgement following the criteria defined by the Barany Society [1]. Currently, there are few validated tools to measure symptom severity or progression through treatment [1].

Clinicians presently use the intensity of visual vertigo (VV), also called visually induced dizziness, as an outcome measure for rehabilitation interventions in people with PPPD. We have previously created a paper version of the Visual Vertigo Analogue Scale (VVAS) [5] to measure VV intensity. The VVAS consists of nine items to rate on a visual analogue scale. Each item pertains to a specific situation that provokes VV [5]. An advantage of using the VVAS is that it is simple and quick to administer [6]. Other questionnaires such as the Dizziness Handicap Inventory (DHI) [7], the Situational Characteristics Questionnaire (SITQ) [8], and the Niigata PPPD questionnaire [9] use ordinal scales to assess VV. With a continuous scale, the VVAS may be more responsive to changes in the intensity of VV post-treatment. However, one major drawback of the VVAS, similar to the questionnaires mentioned above, is the possibility of having recall bias, because the person completing the questionnaire is instructed to rate their symptoms based on their memory of a potentially provoking scenario. People with PPPD tend to avoid provocative situations and activities in daily life, thus, the scoring of the items may not reflect the present reality [10]. To address this, this study proposes to incorporate virtual reality (VR) to improve the assessment of VV in the PPPD population. By recreating symptom-provoking scenarios, a VR assessment tool would allow for immediate rating of VV without relying on delayed recall.

Virtual reality (VR) is defined as “a non-invasive simulation technology that allows a user to interact with a computer-generated environment” [11]. The two modalities used with VR are immersive and non-immersive environments. Non-immersive VR is less interactive and can be as simple as displaying video on a two-dimensional interface [11]. It tends to induce less cybersickness, defined as “symptoms of discomfort and malaise produced by VR exposure” [12]. Thus, this modality may be more accepted by the users [13,14]. Another advantage of a non-immersive system is its relative low cost, leading to a greater utility in clinical settings. For the above reasons, this study proposes the use of a non-immersive VR system, mainly to minimize the risk of adverse events and to ensure that the symptoms experienced can be attributed to the specific scenarios presented to the PPPD population, and not being narrowly linked to the use of VR technology.

The goals of the study were to: (i) develop a non-immersive VR assessment tool inspired from the VVAS for the evaluation of the intensity of VV in people with PPPD; (ii) compare outcomes using the new tool with the original VVAS; (iii) examine feasibility of the tool to distinguish participants with PPPD from healthy controls; and (iv) evaluate the acceptance of the tool.

## 2. Material and Methods

### 2.1. Participants

Eight participants diagnosed with PPPD were recruited from the outpatient vestibular program at the Jewish Rehabilitation Hospital (JRH) in Laval, Quebec. They were matched with eight healthy controls by age and biological sex. Inclusion criteria for the PPPD group were as follows: (i) meet the PPPD diagnostic criteria described by the Barany Society [4]; (ii) corrected vision of 20/30 or better; and (iii) able to attend one in-person session of 60 min at the JRH in July–August 2021. Exclusion criteria included: (i) inability to read English or French independently; (ii) recent acute episode of a vestibular condition; (iii) presence of cognitive impairments limiting ability to complete self-report scales; (iv) history of migraine (control only); and (v) history of vestibular disorder (control only). The study was approved by the ethics committee of the Montreal Centre for Interdisciplinary Research in Rehabilitation. Written informed consent was obtained from all participants.

### 2.2. Development of a Non-immersive Virtual Reality Program

**Figure 1:**
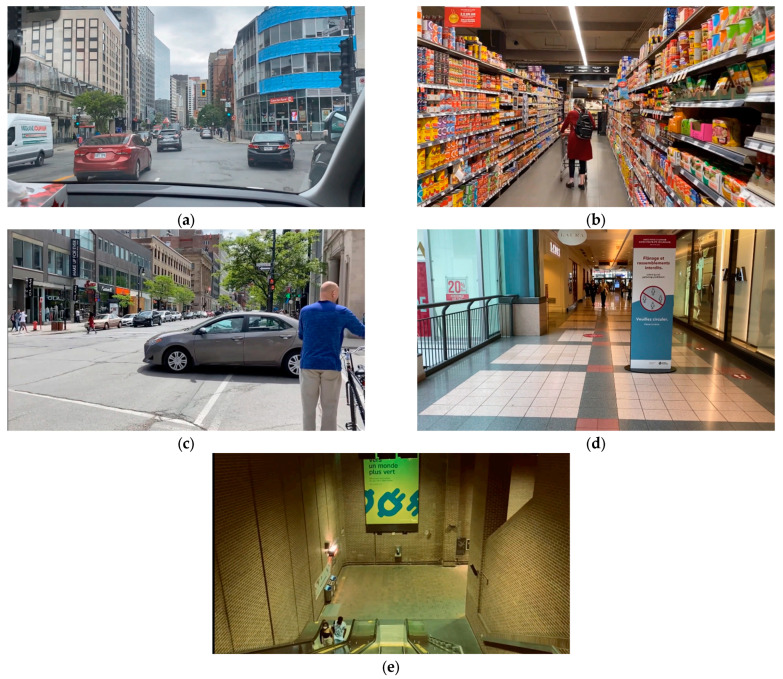
Video scenarios of the c-VVAS: (**a**) being a passenger in a car; (**b**) walking through a supermarket aisle; (**c**) watching traffic at a busy intersection; (**d**) walking through a shopping mall; (**e**) going down an escalator.

Five items from the paper version of the original VVAS (p-VVAS) were adapted into video format of 30 s each to recreate daily situations that provoke vertigo symptom in people with PPPD. The items were (i) walking through a supermarket aisle; (ii) being a passenger in a car; (iii) watching traffic at a busy intersection; (iv) walking through a shopping mall; and (v) going down an escalator (see Figure 1) [5]. The videos were filmed using an iPhone 11 pro max around downtown Montreal. This computerized, video-based VVAS (c-VVAS) included ambient sounds (see Supplementary Video S1–S5) to provide a more immersive experience [15]. The presentation order of the scenarios was randomized using a random sequence generator to avoid order effects.

### 2.3. Assessments

Each participant participated in one 30–60-min in-person session at the research center of the JRH, supervised by a physiotherapist specialized in vestibular rehabilitation (ED). All the assessments were performed at the research center by 2 physiotherapy students. Two other physiotherapy students joined remotely by the Zoom platform (the Zoom sessions were recorded). The participants’ reaction to watching the videos was documented by all the student researchers.

During the session, participants were asked to fill out the original VVAS by marking a vertical line on a 10 cm line to indicate the intensity of their dizziness they recalled experiencing in each of the situations. Distance from the zero endpoint to the marking was measured to the nearest 0.5 mm, and the total p-VVAS score was obtained by adding the score of the 9 items.

Demographic data were collected (age, biological sex, medications, and presence of any anxiety issues) before the assessment using the c-VVAS to provide buffer. Participants were instructed to continue taking their regular medication on the day of the research session. For the PPPD group, information regarding onset of PPPD and interventions received were also collected.

The assessment using the c-VVAS was performed in a quiet room with the lights off. Prior to beginning the c-VVAS evaluation, participants were asked to verbally rate their level of dizziness at that moment (baseline level of dizziness), with 0 being asymptomatic and 10 extreme dizziness. After each scenario, they filled out a visual analogue scale by marking a vertical line on a 10 cm line to indicate the intensity of vertigo they experienced while watching that scenario. Subjects were instructed to look at the crosshair icon displayed on screen and to press the spacebar to start the next scenario once their vertigo returned to baseline level. The scores for each scenario, as well as the mean total c-VVAS score, were obtained the same way as for the p-VVAS (see Supplementary Video S1–S5).

After the assessment using the c-VVAS, participants filled out a questionnaire with a seven 5-point Likert scales (see Supplementary Figure S1), developed based on the technology acceptance model (TAM) [16], to assess the following domains: (i) user’s perceived usefulness of the tool; (ii) perceived ease of use; (iii) attitudes and intention to use. The acceptance rate was calculated by adding the score of each statement (strongly disagree = 0; strongly agree = 4) and transforming the total, which is out of 28, into percentages.

Finally, in an open-question interview, participants commented on positive and negative aspects of the c-VVAS, ways to improve it, and whether they prefer the paper or the computerized version of the VVAS (see Supplementary File S1). The open-question interviews were recorded, and the participants’ answers were transcribed verbatim. An informal process was used to evaluate comments for consistency of positive and negative statements across the subjects. Frequency of common responses was reported.

### 2.4. Data Analysis

Non-parametric statistical analysis using SPSS was performed for this pilot study with small number of subjects. Between-group differences (PPPD vs. control) were determined using Mann–Whitney U test. Pearson correlation coefficient analysis was performed to assess the association of c-VVAS with p-VVAS. Descriptive statistics were used to report demographics and outcomes related to the perception and acceptance of the new technology.

## 3. Results

### 3.1. Participant Characteristics

**Figure 2:**
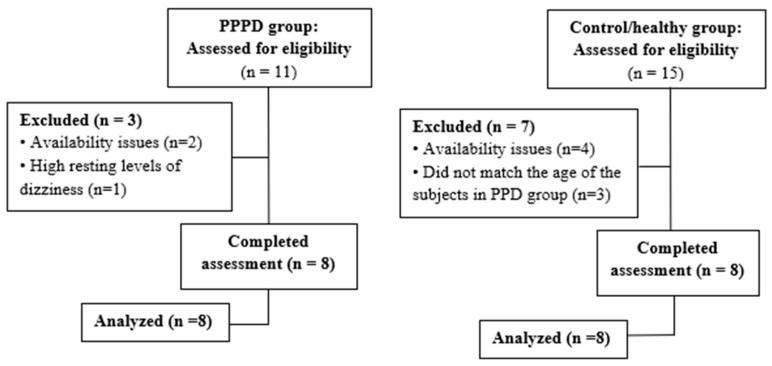
Comparison of group recruitment and participation.

Table 1 illustrates the recruitment process, beginning with the number of subjects assessed in each group, and the final number included in the pilot. The PPPD group consisted of eight subjects, with a mean (SD) age of 53.9 (13.7) years old. Seven (87.5%) were female. For most PPPD subjects (75.0%), the onset of vertigo symptoms was within the last 10 years. Asides from physiotherapy, six (75%) participants received other treatments for their condition, including medications, osteopathy, acupuncture, and psychotherapy. At the time this study was conducted, six of the participants with PPPD were already discharged from the vestibular rehabilitation program of the JRH, yet still had residual dizziness, thus, were qualified for the study. Half of the PPPD group reported no dizziness at baseline, two (25.0%) reported mild dizziness, one (12.5%) reported moderate dizziness, and one reported a high level of dizziness. Almost all participants with PPPD reported issues with anxiety (four (50.0%) with a medical diagnosis and three (37.5%) undiagnosed), similar to what other studies had found [17,18,19]. The control group of eight subjects was matched with the PPPD group by mean age (53.0 ± 13.4) and sex ratio (87.5% female). None reported any dizziness at baseline nor issues with anxiety (Table 1 and Figure 2).

### 3.2. Specificity of the c-VVAS

**Figure 3:**
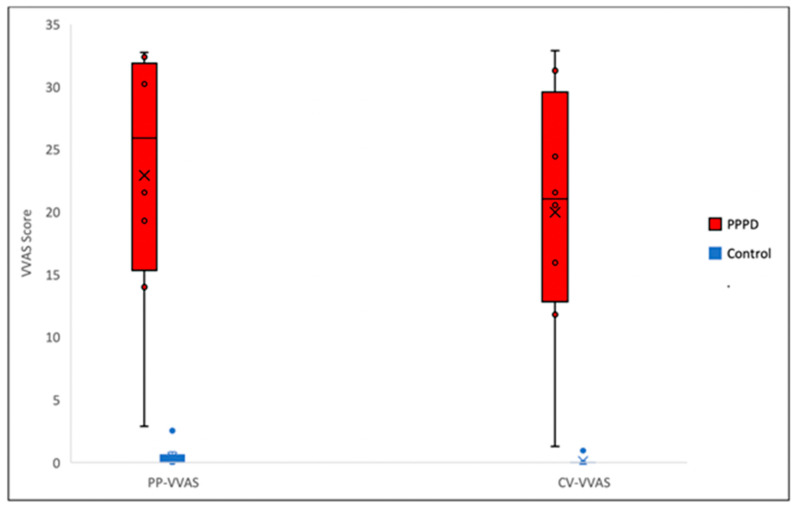
Comparison between the mean scores of dizziness symptoms from PPPD and control participants for both p-VVAS and c-VVAS. (PP-VVAS = paper version of VVAS, CV-VAS=computer version of VVAS).

The Mann–Whitney U test shows a significant difference (*p* = 0.001) between the mean total scores of dizziness symptoms of the PPPD group (22.931) and the control group (0.425), measured by the -p-VVAS. There is also a significant difference (*p* = 0.000) between the mean total scores of c-VVAS of the PPPD group (19.975) and the control group (0.119) (Figure 3, Table 2).

The control subjects reported no dizziness (0/10) watching the scenarios of the -c-VVAS, with only one exception, where the participant reported a very mild level of dizziness provoked by the *passenger in a car*. For each scenario of the -c-VVAS, the Mann–Whitney U test shows a significant difference (*p* < 0.05) between the mean score of the PPPD group and of the control group (Table 3).

### 3.3. Concurrent Validity of the c-VVAS

**Figure 4:**
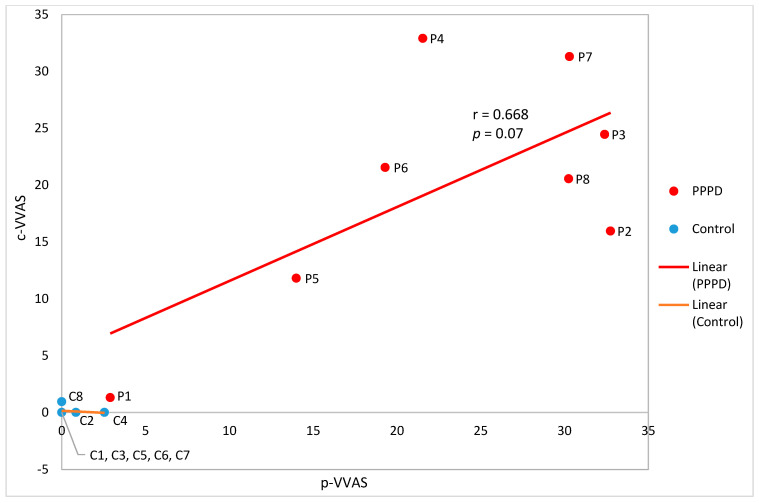
Pearson correlation coefficient to compare the total p-VVAS score with the total c-VVAS score. Each data point corresponds to a pair of p-VVAS and c-VVAS scores rated by one participant.

The Pearson correlation coefficient is r = 0.668 between the total p-VVAS score and the total c-VVAS score obtained from the PPPD group. Both scores measure the intensity of VV symptoms. However, the correlation is not significant (Figure 4). The correlation coefficient for the control group is close to zero.

Out of the five c-VVAS scenarios, only the *escalator* shows a very strong and statistically significant correlation (r = 0.907, *p* < 0.05) with the p-VVAS scores [20]. The correlations for the rest of the scenarios are all non-significant (Table 4).

### 3.4. Acceptance of the c-VVAS

Overall, the study found a high acceptance of this new c-VVAS assessment tool by the participants, with a mean total score of acceptance of 91.74% across the two groups. The domain with the highest score is the perceived ease of use (EOU) for both the PPPD group (98.44%) and the control group (100.00%). The control subjects are more willing to use the c-VVAS (100.00%) in the future compared to the PPPD group (85.94%), whereas the PPPD participants perceive a greater usefulness of the tool (90.63%) compared to the control group (81.25%) (Table 5).

Based on the participants’ feedback regarding the c-VVAS, there was a consensus that the five scenarios were well-chosen and depict well the real-life situations. Another positive comment was the appropriate length of the videos. When asked about ways to improve the c-VVAS, a frequent suggestion was the addition of dynamic tasks, as for this study, participants were seated and stationary. It was also stated that including head and body movements would evoke a more realistic experience. Lastly, two subjects recommended the use of VR headset or a bigger computer screen, to make the experience more immersive.

## 4. Discussion

### 4.1. Specificity of the c-VVAS

The results indicate that the c-VVAS scores, both the total and the sub-scores for each scenario, from the PPPD and the control group are significantly different. This demonstrates that the tool is specific to PPPD population, similar to the p-VVAS [5]. All control subjects rated 0/10 for all scenarios of the c-VVAS, except for one person who reported a very mild level of dizziness in the scenario of passenger in the car. This suggests that this new assessment tool is not inducing cybersickness in people, which is a concern linked to the use of VR technology [12]. This also suggests that symptoms experienced by the PPPD participants are attributable to the specific scenarios presented. It is worth noting that the control subject who reported dizziness with the c-VVAS described the video to be “shaky”, causing her to feel slightly uncomfortable. A possible reason is that the vestibulo–ocular reflex (VOR) is inactive when subjects are stationary and looking straight ahead. The problem of “shakiness” could potentially be solved with the use of a camera stabilizer during the recording.

### 4.2. Correlation of the c-VVAS with p-VVAS

The preliminary data of this pilot study show that the correlation between the total computerized and paper VVAS scores is not significant (*p* = 0.07), with a coefficient of 0.668. Though the correlation between the two tools is not statistically significant, there is still value from the c-VVAS. A benefit of the c-VVAS is it standardizes the visual vertigo image that the responder is rating, removing the recall bias of the p-VVAS. Unifying the visual vertigo stimulus the responder is rating may allow for easier comparison between people and sessions tested. Another benefit is the videos provoke pure visual vertigo; symptoms provoked by balance issues do not bias the answer. The results suggest that c-VVAS is able to elicit the dizziness of participants with PPPD and, thus, provide the necessary construct validity to further test this tool with a large sample size and generate robust results.

Among the five computerized scenarios, only the scores of the *escalator* demonstrate a very strong and significant correlation (r = 0.907, *p* < 0.05) with the scores of the p-VVAS [20]. One explanation is that this scene, in combination with the test condition, i.e., subjects in static sitting, replicate the best situations in real-life, where individuals remain stationary and wait passively to exit the escalator. For scenarios such as the *supermarket*, *shopping mall*, and *busy intersection*, in reality individuals would be in constant motion and interacting with their surroundings. The potential mismatch between what the participants envisioned when filling out the p-VVAS and what was presented in the videos could explain why there were no clear associations found for these scenarios.

### 4.3. c-VVAS Acceptance and Usability

The c-VVAS was highly accepted by all participants, similar to the findings of other studies on the use of VR in various populations [21,22,23]. Participants in both groups strongly agreed that it was easy to use the tool (98.44% and 100%, respectively), suggesting its potential to be implemented in clinical settings as it would be easy for clients to learn to self-administer it. The PPPD group demonstrated a weaker intention to re-use the c-VVAS in the future (85.93%) compared to the control group (100%). This difference seems to stem from the fact that the tool does trigger/increase symptoms in people with PPPD but not in the controls, and that symptoms of VV can be quite uncomfortable. However, the PPPD group perceived the c-VVAS to be more useful (90.63%) compared to the control group’s rating (81.25%). In the post assessment question of which evaluation tool the participants preferred, all participants chose the c-VVAS over the p-VVAS. They explained that the c-VVAS provides a more immersive and real-life experience where they could better envision themselves in the presented situation, which suggests the potential prevention of the recall bias that is associated with the p-VVAS. Indeed, some participants with PPPD were hesitant when filling out the p-VVAS as they had been avoiding the places that provoke their VV. Such avoidant behaviors are common in people with VV [10].

Another benefit of the c-VVAS is that it allows clinicians to document the non-verbal reactions displayed by participants with PPPD while watching the scenarios, which cannot be observed with the p-VVAS. Interestingly, some participants with high p-VVAS and c-VVAS scores showed more signs of discomfort (e.g., looking away, holding the table, squinting their eyes, etc.) with particular c-VVAS scenarios. Having the participant watch specific video clips ensures that the symptom-provoking item being rated is the same for each participant and the same on retesting.

When asked about potential ways to improve the assessment tool, participants mentioned the use of dynamic tasks (e.g., walking, turning, avoiding obstacles, etc.) to make the experience more realistic. However, this would necessitate the use of immersive VR (with the VR headset), which may increase sensory conflicts and, therefore, cybersickness, unless the simulation is very sophisticated and provides synchronized visual–vestibular cues [12,24]. An immersive environment may simply be too provocative for those with a high baseline of VV. Moreover, the equipment required for immersive VR is much more expensive and, thus, unlikely to be practical clinically.

The c-VVAS appeared to be safe as all participants with PPPD completed the five scenarios with no reported adverse effects and no need for clinician’s intervention. Also, based on their feedback, the symptoms triggered seemed to be short-lasting. This suggests that the computerized system is safe for use in a clinical setting.

### 4.4. Limitations and Future Recommendations

Due to the nature of the study, convenience sampling was performed. Also, one person with PPPD refused to participate due to a high intensity of baseline VV. Due to these limitations, in addition to a small sample size, our sample may not be representative of the whole PPPD population, and our results cannot be generalized. Future studies with more participants are suggested.

One drawback is that participants may have remembered what they rated in the p-VVAS when filling out the c-VVAS. A solution would be to add a longer buffer time or randomize the order of testing. Additionally, it is recommended to compare the c-VVAS with other existing and validated questionnaires such as the DHI, the SITQ, and the Niigata PPPD questionnaire [7,8,9]. While this study only evaluated the acceptance of the tool in those with PPPD, it may be more informative to assess acceptance in clinicians as well. Finally, further studies are needed to evaluate the responsiveness of the measure to changes in the intensity of VV symptoms following treatment.

## 5. Conclusions

Even with a small sample of people with PPPD and controls, the information gathered from our end-users of this new computer video-based assessment tool is encouraging in terms of specificity and acceptance. A larger scale study with the inclusion of other recommendations mentioned above could be useful to further investigate the validity of the computerized version. Although the p-VVAS has been validated, recall bias remains a flaw. The purpose of the computerized version is to expose people with PPPD to appropriate visual stimuli in a controlled setting, thereby triggering visual vertigo to a lesser degree as compared to real-life situations. This new tool could be an avenue for health practitioners to track changes in dizziness symptoms over time.

## Figures and Tables

**Table 1:** Demographic data of eight participants experiencing dizziness due to PPPD and eight healthy controls with no dizziness or history of vestibular disorder. (PT—physical therapy treatment).

Demographic Data	Participants			*p*-Value
	PPPD (*n*= 8)	Control (*n* = 8)	Total (*n* = 16)	
Age (year)				0.851 ^1^
Mean (SD)	53.9 ± 13.7	53.0 ± 13.4	53.2 ± 13.5	
Biological sex, *n* (%)				1.000 ^2^
Female	7 (87.5%)	7 (87.5%)	14 (87.5%)	
Time of onset of PPPD, *n* (%)				
1–10 years ago	6 (75.0%)	n/a	6 (75.5%)	
40–50 years ago	2 (25.0%)	n/a	2 (25.0%)	
Baseline dizziness level/10, *n* (%)				
0	4 (50.0%)	8 (100.0%)	12 (75.0%)	
1–4	2 (25.0%)	0 (0.0%)	2 (12.5%)	
5–7	1 (12.5%)	0 (0.0%)	1 (6.25%)	
8–10	1 (12.5%)	0 (0.0%)	1 (6.25%)	
PT Treatment at the JRH, *n* (%)				
Currently under treatment	2 (25.0%)	n/a	2 (25.0%)	
Discharged	6 (75.0%)	n/a	6 (75.0%)	
Other treatment received, *n* (%)				
None	2 (25.0%)	n/a	2 (25.0%)	
Medication	3 (37.5%)	n/a	3 (37.5%)	
Osteopathy	2 (25.0%)	n/a	2 (25.0%)	
Multiple modalities (meds+ acupuncture + osteopathy + psychotherapy)	1 (12.5%)	n/a	1 (12.5%)	
Anxiety, *n* (%)				
None	1 (12.5%)	8 (100.0%)	9 (56.25%)	
Diagnosed	4 (50.0%)	0 (0.0%)	4 (25.0%)	
Undiagnosed	3 (37.5%)	0 (0.0%)	3 (18.75%)	

^1^ Unpaired *t*-test *p*-value, ^2^ chi-square *p*-value.

**Table 2:** Comparison between the mean total scores of the participants in the PPPD group and the control group for the p-VVAS and the c-VVAS using Mann–Whitney Test.

PPPD vs. Control	Mean Score of PPPD (*n* = 8)	Mean Score of Control (*n* = 8)	Mann–Whitney U Coefficient	*p*-Value
p-VVAS	22.931	0.425	0.000	0.001
c-VVAS	19.975	0.119	0.000	0.000

**Table 3:** Comparison between the mean scores of dizziness symptoms of the PPPD group and the control group for each scenario of the c-VVAS using the Mann–Whitney Test.

Scenario	Mean Score of PPPD (*n* = 8)	Mean Score of Control (*n* = 8)	Mann–Whitney U Coefficient	*p*-Value
Supermarket	4.356	0.000	8.000	0.007
Passenger in a car	5.144	0.119	1.000	0.001
Busy intersection	2.938	0.000	4.000	0.001
Shopping mall	3.500	0.000	4.000	0.001
Escalator	4.038	0.000	8.000	0.004
Total	19.975	0.119	0.000	0.000

**Table 4:** Pearson correlation coefficients, between the scores on the p-VVAS and the c-VVAS for each scenario. Data obtained from PPPD group.

Scenario	Pearson Correlation Coefficient (r)	*p*-Value
Supermarket	0.663	0.073
Passenger in a car	0.444	0.270
Busy intersection	0.593	0.121
Shopping mall	0.032	0.941
Escalator	0.907	0.005

**Table 5:** Participants’ perception and acceptance of the c-VVAS, ease of use (EOU). (*) Note that the total score represents a weighted average, with the domain of perceived usefulness being the most important as it includes 3 statements out of 7.

Category	Mean Score of PPPD (%) (*n* = 8)	Mean Score of Control (%) (*n* = 8)	Mean Score of Both Groups (%) (*n* = 16)
Perceived EOU (statement 1, 2)	98.44	100.00	99.22
Attitude and intention to use (statement 3, 4)	85.94	100.00	92.97
Perceived usefulness (statement 5, 6, 7)	90.63	81.25	85.94
Total score (*)	91.52	91.96	91.74

## Data Availability

No new data is available.

## Supplementary Materials

The following supporting information can be downloaded at: https://www.mdpi.com/article/10.3390/jcm12051766/s1.
