# Supplementary material for: A Pilot Study to Assess Visual Vertigo in People with Persistent Postural–Perceptual Dizziness with a New Computer-Based Tool
Source: J Clin Med. 2023 Feb 22;12(5):1766. doi: 10.3390/jcm12051766 (PMC10003047; doi:10.3390/jcm12051766)
Supplement: Supplementary file 1 [file jcm-12-01766-s001.zip › jcm-2230554-supplementary.docx]

**Supplementary Video S1–S5.** Five video files of the c-VVAS.

**Supplementary Figure S1.** Technology Acceptance Questionnaire (TAM).


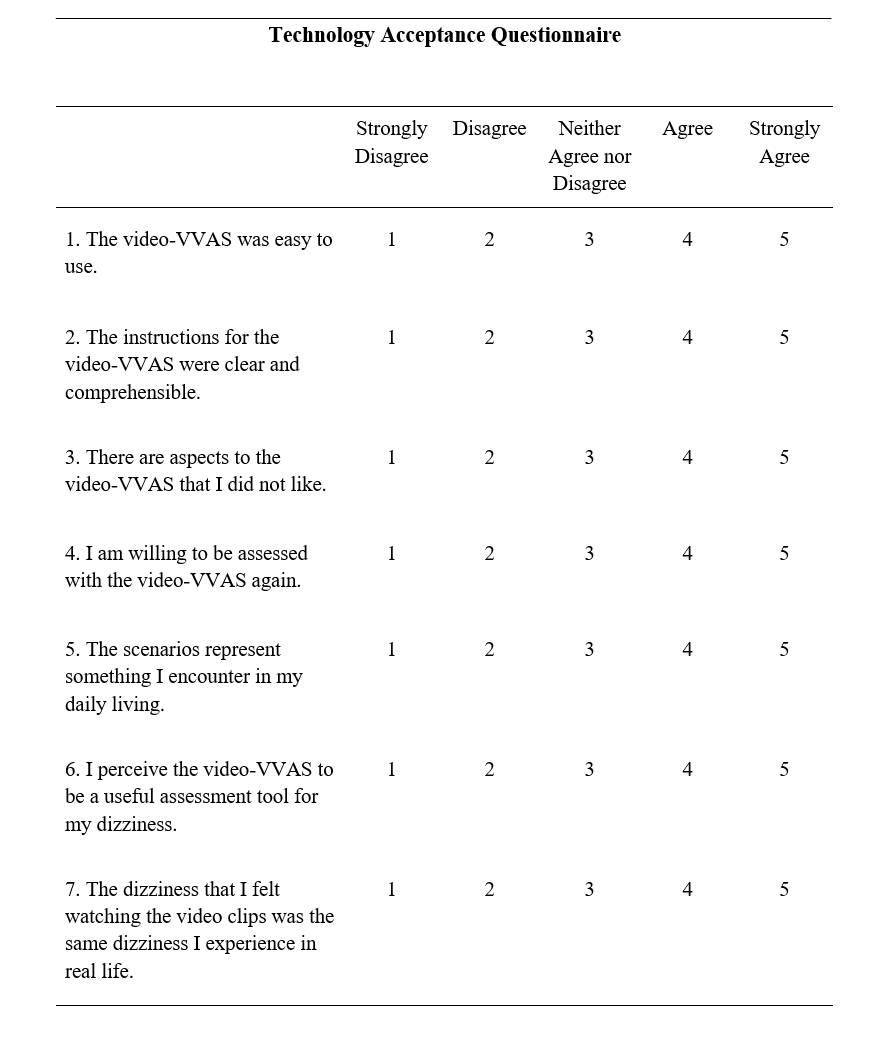


Supplementary File S1. Post session open-ended questions

1a. What did you like about using the computer-based system?

1b. Was there anything you didn’t like?

2. Is there anything you would improve about the system if possible?

3. If you had the choice would you prefer to use the paper-and-pencil version or the computerized version of the Visual Vertigo Analogue Scale (VVAS) to assess your PPPD?
